# CONNECTING HEALTHY BRAINS AND HEALTHY HEARTS IN INDIAN COUNTRY

**DOI:** 10.1093/geroni/igz038.1338

**Published:** 2019-11-08

**Authors:** Kelsey M Donnellan

**Affiliations:** Association of State and Territorial Health Officials (ASTHO), Washington, D.C., United States

## Abstract

The release of the Healthy Brain Initiative Road Map for Indian Country inspired the International Association for Indigenous Aging (IA2), Association of State and Territorial Health Officials (ASTHO), and the Centers for Disease Control and Prevention (CDC) to develop health communication materials to promote heart health and brain health among states with American Indian and Alaska Native (AI/AN) communities. IA2 engaged public health, tribal health, and brain health experts to inform the key messages and intended audiences. The final package includes two posters, one flyer, one provider guide, four radio public service announcements, and two short videos. Tribal health officials and state health are encouraged to distribute the resources through senior centers and inter-tribal organizations, healthcare facilities, administrative offices, tribal newspapers/radio stations, and as mailers to tribal members. The session will focus on how the resources raise awareness of and promote action on heart and brain health among AI/AN communities.

